# The clinical characteristics and prognostic factors of combined Hepatocellular Carcinoma and Cholangiocarcinoma, Hepatocellular Carcinoma and Intrahepatic Cholangiocarcinoma after Surgical Resection: A propensity score matching analysis

**DOI:** 10.7150/ijms.50883

**Published:** 2021-01-01

**Authors:** Youyin Tang, Lingyan Wang, Fei Teng, Tao Zhang, Yunuo Zhao, Zheyu Chen

**Affiliations:** 1 Department of Liver Surgery, Liver Transplantation Center, West China Hospital of Sichuan University, No. 37 GuoXue Alley, Chengdu 610041, P.R. China.; 2 Department of Obstetrics and Gynecology Nursing, Key Laboratory of Birth Defects and Related Disease of Women and Children, West China Second University Hospital, Sichuan University, No. 20 South Renmin Road, Chengdu 610041, P.R. China.; 3 Department of Biotherapy, West China Hospital and State Key Laboratory of Biotherapy, Sichuan University, No. 37 GuoXue Alley, Chengdu 610041, P.R. China.

**Keywords:** combined hepatocellular carcinoma and cholangiocarcinoma, surgical resection, clinical features, long-term survival, propensity score matching

## Abstract

**Background:** Clinical characteristics and prognosis among combined hepatocellular carcinoma (HCC) and cholangiocarcinoma (cHCC-CC) with HCC and intrahepatic cholangiocarcinoma (ICC) were inconsistent in previous studies. The aim of this study was to compare postoperative prognosis among cHCC-CC, HCC and ICC, and investigated the prognostic risk factor of cHCC-CC after surgical resection.

**Methods:** A total of 1041 eligible patients with pathological diagnosis of cHCC-CC (n=135), HCC (n=698) and ICC (n=208) were enrolled in this study. Univariate and multivariate Cox analysis were applied for assessing important risk factors. cHCC-CC were further 1:1 matched with HCC and ICC on important clinical risk factors. Survival curves of matched and unmatched cohorts were depicted by Kaplan-Meier method with log-rank test.

**Results:** Patients with cHCC-CC had similar rate of sex, age and cirrhosis with HCC (*p<*0.05) and comparable incidence of hepatitis B or C with ICC (*p=*0.197). Patients of cHCC-CC had intermediate prognosis between HCC and ICC, with median overall survival (OS) time of cHCC-CC, HCC and ICC of 20.5 months, 35.7 months and 11.6 months (*p<*0.001). In matched cohorts, the OS of cHCC-CC were worse than HCC (*p<*0.001) but comparable with ICC (*p=*0.06), while the disease-free survival (DFS) of cHCC-CC was worse than HCC but better than ICC (*p<*0.05). And lymph node infiltration and postoperative transarterial chemoembolization (TACE) were independent risk factors of cHCC-CC associated with prognosis.

**Conclusion:** The long term survival of cHCC-CC was worse than HCC but comparable with ICC when matched on albumin level, tumor size, lymph node infiltration, tumor stage and margin. Presence of lymph node infiltration and no postoperative TACE were associated with poor prognosis of cHCC-CC.

## Introduction

Combined hepatocellular carcinoma (HCC) and cholangiocarcinoma (cHCC-CC) is a relatively rare type of liver tumor, with an incidence of 0.8%-6.5% in primary liver carcinoma 1-6. Considering of this special phenotype of primary liver malignancy, several medical terms, such as mixed HCC and cholangiocarcinoma or dual-phenotype HCC, were applied to describe cHCC-CC for decades 7, 8. In recent years, a valuable nomenclature by World Health Organization (WHO) defined cHCC-CC as a single nodule with both differentiation of hepatocellular carcinoma (HCC) and intrahepatic cholangiocarcinoma (ICC), and removed the subtype of cholangiolocellular carcinoma (CLC) which was considered as a subtype of cHCC-CC in the 4^th^ edition 9, 10.

Albeit the clinical characteristics and prognosis of among cHCC-CC, HCC and ICC had been widely discussed for years, the results yielded in their studies were still inconsistent 2, 4, 6, 11-14. Most of the previous studies found that cHCC-CC had worse prognosis than HCC 2, 4-6, 12, 15-19, while some of them suggest the prognosis of cHCC-CC was better than ICC 2, 4, 6, and others insisted opposite conclusion 5, 12, 15-19. In the literature review, large-scale studies regarding to the long-term outcome among cHCC-CC, HCC and ICC were limited and a recently western series which comprised 623 patients found that no significant difference of 5-year survival rate of cHCC-CC, HCC as well as ICC after surgical resection 11. However, they only included 47 cHCC-CC patients. Thus, the conclusions yielded in their studies were still limited, meaning the clinical characteristics and prognosis of cHCC-CC compared to HCC or ICC were still inconclusive.

In the current study, we aimed to compare the clinical features and long term survival of cHCC-CC compared with HCC and ICC after surgical resection, identifying survival risk factors of cHCC-CC after surgical resection.

## Materials and Methods

### Patient selection

This study was approved by the Ethics Committee of West China Hospital of Sichuan University. And inform consent was waived because no individual information was disclosed in this study. The medical records of patients diagnosed with cHCC-CC, HCC and ICC during January 2012 to June 2017 at West China Hospital were retrospectively reviewed. The diagnosis of cHCC-CC abided by World Health Organization classification of digestive system tumours, and cholangiolocellular carcinoma (CLC) were excluded in this study 9, 10. HCC with expression of keratin (K) 19 was also excluded since the distinction of K19 positive HCC and cHCC-CC was ambiguous 20.

**Figure 1:**
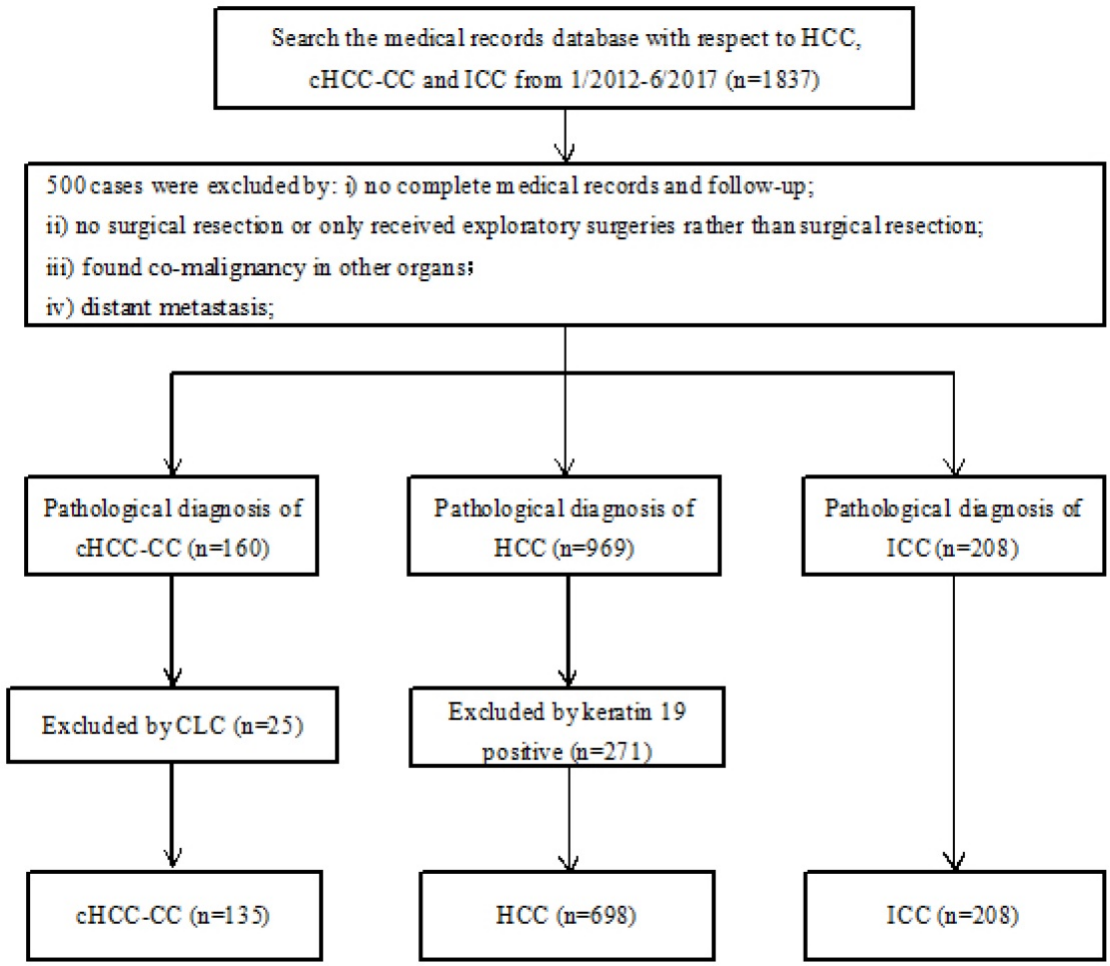
Flow diagram of this study. Abbreviations: HCC: hepatocellular carcinoma; cHCC-CC: combined hepatocellular carcinoma and cholangiocarcinoma; ICC: intrahepatic cholangiocarcinoma; CLC: cholangiolocellular carcinoma.

The additional inclusion and exclusion criteria were: Inclusion criteria: i) patients had complete medical records and follow-up; ii) patients had pathological diagnosis of cHCC-CC, ICC and K19 negative HCC according to the latest WHO classification; Exclusion criteria: i) patients only received exploratory surgeries rather than surgical resection; ii) patients had distant metastasis (M1 stage); iii) patients had co-malignancies in other organs. **Figure 1** was the patient selection flow chart.

### Study design and propensity score matching

In order to minimize the impact of distinguishing clinical characteristics among cHCC-CC, HCC and ICC patients, patients were further enrolled into two separate matched cohorts: i) cHCC-CC were 1:1 matched with HCC on albmin (ALB) level, tumor size, lymph node (LN) infiltration, 8^th^ American Joint Committee on Cancer (AJCC) staging and margin; ii) cHCC-CC were 1:1 matched with ICC regarding to ALB level, tumor size, lymph node infiltration, 8^th^ AJCC staging and margin.

### Preoperative management and surgery

All patients' baseline characteristic and surgical details as well as pathological features were carefully reviewed. Enhanced imaging examination including ultrasonography, computed tomography, magnetic resonance imaging were used to preoperatively assess the tumor size, location, invasion of portal vein, bile duct or hepatic arteries and presence of intro-hepatic or extra-hepatic metastases in all patients. Patients with Child-Pugh class A liver function and performance status score less than 2 were eligible for surgical resection. Surgical methods were assessed by preoperative multidisciplinary team discussion. In the present study, hypersplenia was diagnosed by: i) enlarged spleen (thickness of spleen was more than 4cm in ultrasonography); ii) peripheral cytopenias, including the decreasing of red blood cells, white blood cells and platelets. Major liver resection was defined as more than two segments resection while the minor liver resection was considered as ≤2 segments resection. All enrolled patients had routine follow-up at first month and every 6 month subsequently until May 2020. The primary endpoint of this study was overall survival (OS) and OS was calculated from the time receiving surgery to the time of death or May 2020. The second end point was disease-free survival (DFS) which was defined as the duration between surgical resection and recurrence or metastasis.

### Statistical analysis

Power analysis was performed to ensure sufficient subjects in this study. An estimated 695 patients would be needed to provide 90% power for 5-year overall survival log-rank test with a two-sided α of 0.05 (Supplement Table 3). To compare baseline demographic and clinicopathologic characteristics among patients diagnosed with cHCC-CC, HCC and ICC, Kruskal-Wallis test (for continuous variables) as well as χ^2^ test and Fisher exact tests (for categorical variables) were all used in this study. Within the unmatched cohort and matched cohorts, survival curves were depicted using Kaplan-Meier methods with comparison of log-rank test. Univariate analysis and multivariate Cox regression analysis with step-wise selection were used to compare statistical difference of overall survival in unmatched and matched cohort. Variables with *p* value <0.1 in univariate analysis were further selected in multivariate Cox regression analysis. A *p*-value <0.05 was considered statistically significant. All statistical analyses were conducted with EmpowerStats software, version 2.20.

## Results

### Patient characteristics

A total of 1041 patients were included in our study, with 135 patients in cHCC-CC group, 698 in HCC group and 208 in ICC group. Among them, there were 847 males, accounting for 81.4% of the whole cohort. In addition, 776 patients (74.5%) were diagnosed at age<60 years. Surgical resection was applied for most patients (98.3%), while anatomy resection was performed in 467 cases. Patients' baseline characteristics and pathological details were summarized in **Table 1.**

cHCC-CC patients tended to have more similar baseline clinical features with HCC, such as hypertension, diabetes mellitus, alanine aminotransferase (ALT) level, aspartate aminotransferase (AST) level, ALB level, total bilirubin level. The rate of liver cirrhosis in cHCC-CC patients was comparable with HCC (cHCC-CC versus HCC, 40.7% versus 51.0%, *p=*0.32), yet higher than that in ICC patients (cHCC-CC vs. ICC, 50.4% vs. 43.3%, *p<*0.01). The incidence of hepatitis B or C of cHCC-CC was similar with ICC (cHCC-CC vs. ICC, 50.4% vs. 43.3%, *p=*0.197), but significantly lower than that of HCC (cHCC-CC vs. HCC, 50.4% vs. 73.9%, *p<*0.001). Considering of collinearity between vascular invasion and tumor thrombus, we only included the tumor thrombus data of each patient, and micro vascular thrombus was also included as tumor thrombus 21. And we found that the incidence of tumor thrombus of cHCC-CC (37.0%) was higher than ICC (21.6%) but less than HCC (51.1%). And this was compared to previous study, which reported the incidence of vascular invasion was about 9% to 89.5% in cHCC-CC 4, 16, 22.

cHCC-CC had higher rate of advanced tumor stage (AJCC stage III+IV) than HCC and ICC (68.1% in cHCC-CC vs. 50.7% in HCC and 46.1% in ICC, *p<*0.001). ICC patients had higher rate of advanced tumor grade (poor or undifferentiated grade) than cHCC-CC and HCC patients (69.2% in ICC vs. 53.3% in cHCC-CC and 51.5% in HCC, *p<*0.05). No significant difference was found in tumor size among three groups (*P=*0.55).

### Survival analysis and prognosis predictors prior to match

**Figure 2:**
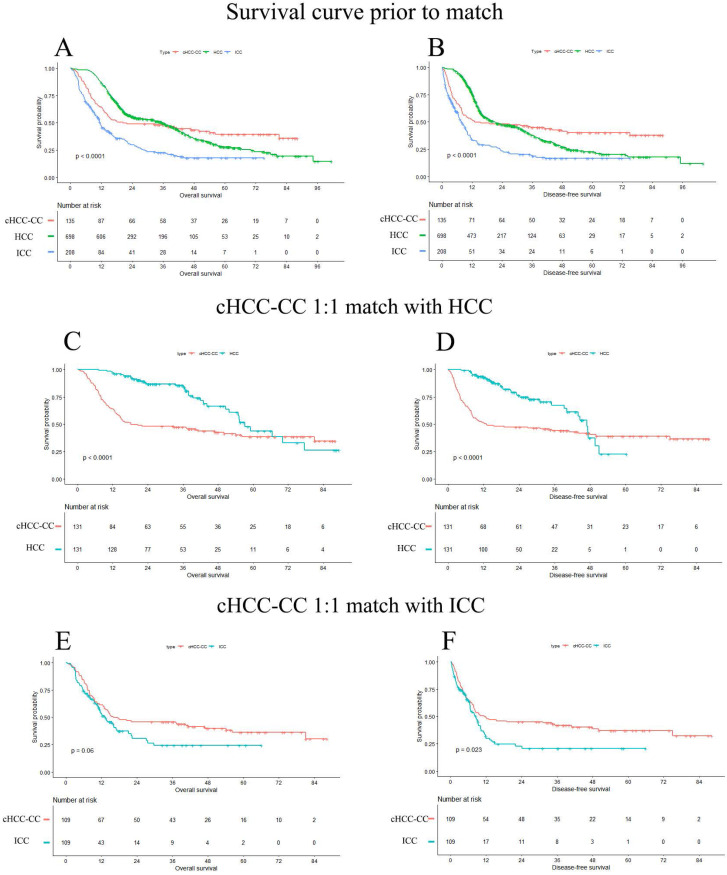
Survival curves prior to match and matched cohorts. **A and B:** OS and DFS of unmatched cohort. **C and D:** OS and DFS in cHCC-CC 1:1 matched with HCC cohort. **E and F:** OS and DFS in cHCC-CC 1:1 matched with ICC cohort. The median overall survival time prior to match of cHCC-CC, HCC and ICC was 20.5months, 35.7 months and 11.6 months (*p<*0.001), respectively. The prognosis of cHCC-CC were comparable with ICC (*p=*0.0599), yet worse than HCC after match (*p<*0.001). OS: overall survival; DFS: disease-free survival; cHCC-CC: Combined hepatocellular carcinoma and cholangiocarcinoma; HCC: hepatocellular carcinoma; ICC: intrahepatic cholangiocarcinoma.

Until May 2020, a total of 624 (59.9%) patients died and the median follow-up time was 26.8 months, with 95% confidence interval (CI) of 22.1 to 31.6 months. Overall, the 1- and 3-year OS rate of cHCC-CC, HCC and ICC were 63.9%, 86.7%, 47.2% and 48.1%, 49.8%, 22.6%, respectively, and 5-year OS rate of cHCC-CC, HCC and ICC was 39.5%, 27.8% and 17.9%, respectively. The median OS time of cHCC-CC, HCC and ICC was 20.5 months, 35.7 months and 11.6 months, respectively (*p<*0.0001). The survival curve among cHCC-CC, HCC and ICC group prior to match was showed in **Figure 2A and B.**

In multivariate Cox analysis, we found ALB level, tumor size > 5 cm, lymph node infiltration, advanced AJCC stage (III and IV), advanced differentiation grade (poor grade and undifferentiated grade), positive margin, postoperative TACE and tumor type were important risk factors associated with poor prognosis. Besides, we found cHCC-CC had similar prognosis to HCC (HR=1.1, 95% CI: 0.8-1.4, *p=*0.72), but had better prognosis than ICC (HR=3.5, 95% CI: 2.6, 4.8, *p<*0.001). The univariate analysis and multivariate Cox analysis prior to match was showed in **Table 2** and more details of univariate analysis and multivariate Cox analysis prior to match was displayed in Supplement Table 1.

### Survival analysis and prognosis predictors after matching on ALB level, tumor size, lymph node infiltration, AJCC stage and margin

Since most of the risk factors found in unmatched cohort except tumor size were significantly different among cHCC-CC, HCC and ICC group, we conducted a further analysis by two separated matched cohorts (cHCC-CC 1:1 matched with HCC; and cHCC-CC 1:1 matched with ICC) using propensity score matching on ALB level, tumor size, lymph node infiltration, AJCC stage, differentiation, margin and postoperative TACE. After matching, most of the above variables were found no significant difference in two matched cohorts, except for differentiation (cHCC-CC versus HCC, *p=*0.0028; cHCC-CC versus ICC, *p=*0.016), margin (cHCC-CC versus HCC, *p=*0.046) and postoperative TACE (cHCC-CC versus HCC, *p<*0.0001) (**Table 3**).

After matching, survival curves regarding to OS and DFS of two matched cohorts were presented in **Figure 2.** The OS of cHCC-CC were significantly worse than HCC (*p<*0.001) but comparable to ICC (*p=*0.0599). The median OS time of two cohorts were (cHCC-CC versus HCC, 18.2 months versus 57.3 months, *p<*0.001; cHCC-CC versus ICC, 16.1 months versus 13.1 months, *p=*0.06). The DFS of cHCC-CC were significantly worse than HCC (*p<*0.001), however better than ICC (*p=*0.0229). The median DFS time of two cohorts were (cHCC-CC vs. HCC, 13.0 months versus 46.9 months, *p<*0.001; cHCC-CC vs. ICC, 11.3 months versus 8.6 months, *p=*0.0229).

In multivariate Cox analysis of matched cohorts, we found positive lymph node infiltration (HR: 2.5, 95%CI: 1.1-5.0, *p=*0.025) and no postoperative TACE (HR: 2.8, 95%CI: 1.8- 4.3, *p<*0.001) were relating to poor prognosis in both cHCC-CC 1:1 matched HCC cohort and cHCC-CC 1:1 matched ICC cohort. The univariate analysis and multivariate Cox regression analysis of two matched cohorts were showed in **Table 4.**

**Figure 3:**
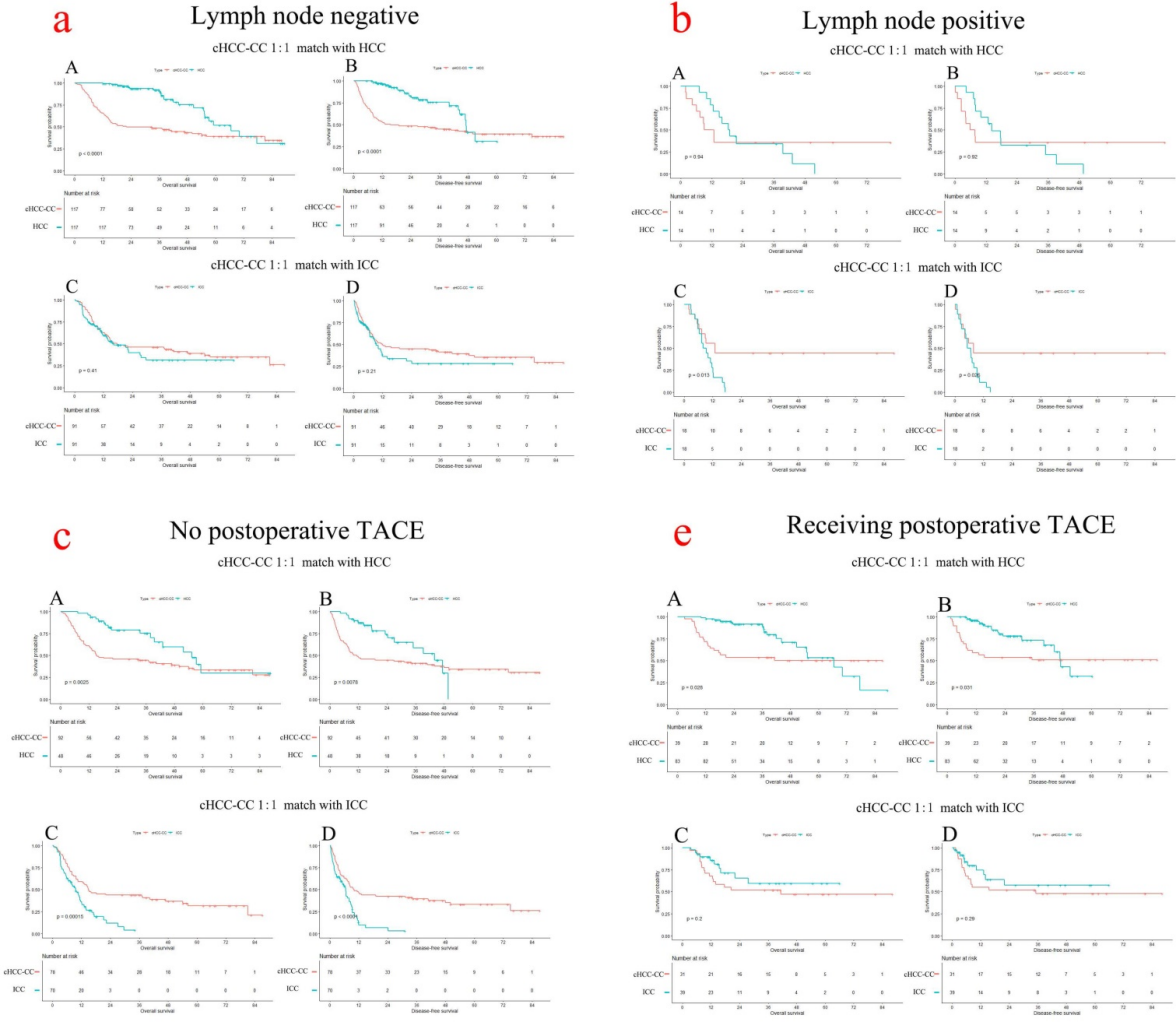
Survival curves of matched cohorts stratified by LN infiltration and postoperative TACE. **A-D:** The OS and DFS in cHCC-CC 1:1 matched with HCC cohort and cHCC-CC 1:1 matched with ICC cohort, respectively. a and b: Survival curves of matched cohorts stratified by LN infiltration; c and d: Survival curves of matched cohorts stratified by postoperative TACE. cHCC-CC had significantly worse prognosis than HCC in LN negative patient (*p<*0.001) but better prognosis than ICC in LN positive patient (*p<*0.05). cHCC had significant worse prognosis than HCC in either receiving or not receiving postoperative TACE patients (*p<*0.05), but have better prognosis than ICC in no postoperative TACE patients (*p<*0.001). LN: lymph node; TACE: transhepatic arterial chemotherapy and embolization; OS: overall survival; DFS: disease-free survival; cHCC-CC: Combined hepatocellular carcinoma and cholangiocarcinoma; HCC: hepatocellular carcinoma; ICC: intrahepatic cholangiocarcinoma.

While stratified by negative lymph node infiltration of two matched cohorts, cHCC-CC patients had significantly poor overall survival and disease-free survival than HCC patients (OS: cHCC-CC vs. HCC, *p<*0.001) but similar to ICC (OS and DFS, cHCC-CC vs. ICC, *p=*0.41 and *p=*0.21, respectively) (**Figure 3a**). However, when stratified by positive lymph node infiltration of two matched cohorts, the OS rate and DFS rate of cHCC-CC were similar to HCC but better than ICC (**Figure 3b**). The median overall survival time of cHCC-CC stratified by lymph node (LN) infiltration were significantly different (median overall survival time, LN positive vs. LN negative: 22.1 months vs. 12.9 months, *p=*0.019) (Supplement Figure 1A).

In no postoperative TACE patients of two matched cohorts, the prognosis of cHCC-CC were better than ICC (median overall survival time, cHCC-CC vs. ICC: 15.0 months vs. 9.6 months, *p=*0.0002) but worse than HCC (median overall survival time, cHCC-CC vs. HCC: 15.2 months vs. 55.4 months, *p=*0.0025) (**Figure 3c**). In patients receiving postoperative TACE of two matched cohorts, cHCC-CC had significantly poor overall survival than HCC (*p=*0.028) but similar to ICC (*p=*0.2) (**Figure 3d**). The mean overall survival time of cHCC-CC stratified by postoperative TACE were significantly different (mean overall survival time, TACE vs. no TACE: 52.0 months vs. 35.2 months, *p=*0.012) (Supplement Figure 1C).

## Discussion

cHCC-CC is a distinct entity of primary liver tumor, with a proportion of about 0.8-6.5% in primary liver malignancy 2, 6, 15, 17, 18. However, the results of previous studies regarding to outcomes of cHCC-CC were inconsistent. In the present study, we found the long-term prognosis of cHCC-CC was worse than HCC, however, better than ICC in the whole cohort, but the long term overall survival of cHCC-CC was worse than HCC, yet similar to ICC in matched cohorts. Besides, the multivariate cox regression analysis within matched cohorts revealed that the independent prognosis risk factors of cHCC-CC were lymph node infiltration and postoperative TACE.

The clinical characteristics and prognosis of cHCC-CC compared to HCC and ICC were inconsistent, albeit it had been discussed for decades. In this study, we found baseline clinical characteristics of cHCC-CC were comparable with HCC regarding to age, gender, and incidence of hypertension, diabetes mellitus and cirrhosis. These findings were in consistent with previous studies which found a similar incidence of demographic characteristics and cirrhosis between cHCC-CC and HCC 4, 11, 12, 23. Besides, the incidence of hepatitis B or C of cHCC-CC was similar to ICC (*p=*0.197) but different from HCC (*p<*0.001). This was in consistent with Jarnagin et al. which found that the rate of hepatitis B or C between cHCC-CC and ICC was similar, with an incidence of 15% in cHCC-CC patients 5. cHCC-CC showed an intermediate prognosis between HCC and ICC prior to match (median overall survival time: cHCC-CC, HCC and ICC, 20.5 months, 35.7 months and 11.6 months, *p<*0.001), and a comparable prognosis with ICC, yet worse than HCC in matched cohorts (median overall survival time: cHCC-CC versus ICC, 16.1 months versus 13.1 months, *p=*0.06; cHCC-CC versus HCC, 18.2 months versus 57.3 months, *p<*0.001). This can be demonstrated by previous study which reported the prognosis of cHCC-CC was worse than HCC, however, comparable with ICC, with median overall survival time of 6.0 months, 17.4 months, and 4.4 months in cHCC-CC, HCC and ICC, respectively 12. Another population‐based study suggested that the 5-year OS rate of cHCC-CC, HCC and ICC was 34.4%, 43.5% and 33.3%, respectively 15. The clinical characteristics and prognosis of cHCC-CC were different from HCC and ICC, suggesting that cHCC-CC was a distinct entity of primary liver malignancy and should be treated individually.

Lymph node infiltration was associated with poor survival of cHCC-CC after surgery 15, 24. In the present study, the incidence of lymph node infiltration in cHCC-CC was 13.3% and patients with positive LN infiltration survived significant poor prognosis than those negative (median overall survival time, positive vs. negative, 22.1 months vs. 12.9 months, *p=*0.019). Even in 1:1 matched cohort with HCC, cHCC-CC still had poor prognosis than HCC either in LN negative (median overall survival time, cHCC-CC vs. HCC, 22.1 months vs. 66.6 months, *p<*0.001) or LN positive patients (median overall survival time, cHCC-CC vs. HCC, 11.1 months vs. 18.7 months, *p=*0.937) (**Figure 3a, 3b**). Previous study found the incidence of lymph node metastasis in cHCC-CC was about 8.3%-60.0% 6, 13, 14, 25, with an average rate of 48% 26. In further, cHCC-CC patients with positive LN infiltration suffered an unfavorable average overall survival time of 7.8 months than those of 20.2 months in negative LN 13. Another population-based study suggested that lymph node status of cHCC-CC were strongly associated with overall survival, with a remarkable increased risk of death in positive LN patients 15. A recent systematic review of cHCC-CC revealed that LN metastasis was strongly associated with decreased overall survival after surgical resection, with a hazards ratio of 2.84, *p<*0.0001 26. As LN metastasis resulted in worse prognosis than negative LN and lymphadenectomy was only performed in about one third of cHCC-CC patient 15, lymphadenectomy should be regarded as a routine procedure for suspected cHCC-CC following curative surgery.

Postoperative TACE may benefit for OS and DFS for cHCC-CC after surgical resection. In this study, although cHCC-CC patients obtain significant worse prognosis than HCC patients in either receiving or not receiving postoperative TACE (*p<*0.05), cHCC-CC patients who received postoperative TACE still obtained a favorable prognosis than those who didn't (mean overall survival time, TACE vs. no TACE: 52.0 months vs. 35.2 months, *p=*0.012). TACE was considered as an alternative treatment method of advanced or unresectable HCC or an adjuvant therapy following surgical resection 27-29. Previous studies reported that TACE served as an adjuvant method which prolonged survival of HCC following surgical resection, with a 5-year OS rate of 53.3% in patients receiving more than twice TACE treatments 30. Moreover, Kim et al. reported that TACE could significantly improve the OS of unresectable cHCC-CC, with a favorable median OS time of 12.3 months 31. As we know, tumor hypovascularity was the major factor restricting the response of TACE 24, 31. Although some studies insisted that cHCC-CC was hypovascular and TACE was not recommended 32, 33, the rate of hypervascularity in cHCC-CC in previous study reached about 40.9%-80% 31, 34, which enabled a favorable feasibility and effect of applying postoperative TACE for cHCC-CC. Thus, more studies regarding to the proportion of tumor arterialization could be conducted to verify the effect of TACE in cHCC-CC.

Sorafenib therapy, as an alternative treatment method, was used in many advanced primary liver malignancies. A recent systematic review showed that sorafenib was not superior to hepatic arterial infusion chemotherapy in advanced HCC patients, because that sorafenib was associated with diarrhea and hand-foot syndrome 35. In addition, capecitabine was recommended as an alternative adjuvant chemotherapy following surgical resection of biliary tract cancer 36. As for cHCC-CC, only one study that reported the use of molecular targeted therapy, but failed to demonstrate the superiority of molecular targeted therapy in terms of recurrence or survival 37. In the present study, we found that only few patient (<5%) received sorafenib treatment after surgical resection and whether the others were being treated by sorafenib was unknown. So we didn't include this variable in the study. This was also a limitation resulting from the nature of retrospective study.

The study has several strengths. First, this study enrolled a large cohort of patients with long-term follow-up and histological evidence. Second, the definition of cHCC-CC in most of the previous study was vague; however, in the present study, we adopted the latest classification (2019 WHO) and removed patients who were diagnosed as cholangiolocellular carcinoma (CLC). Third, we applied propensity score matching analysis, to reduce the bias of potential confounding in baseline characteristics and the groups were well matched. Forth, although the benefit of TACE for cHCC-CC remained debatable in many previous studies,^31^-^33^ this study found that postoperative TACE could benefit for survival for cHCC-CC after surgical resection.

Two limitations should be mentioned in this study. First, the nature of retrospective study may generate selective bias. Second, although the intention of propensity score matching of baseline variables was to reduce differences between groups, a decreased sample size will appear when increasing matched variables. Thus, in this study, we only matched variables relating to prognosis and most of cHCC-CC patients were enrolled in two matched cohorts, and only few cHCC-CC patients were excluded in matched cohorts.

## Conclusion

The baseline clinical features and prognosis of cHCC-CC were inconsistent with HCC and ICC, and should be considered as a distinct entity of primary liver malignancy. The long term survival of cHCC-CC was worse than HCC, yet comparable with ICC when matched on ALB level, tumor size, lymph node infiltration, AJCC stage and margin. Presence of LN infiltration and no postoperative TACE were associated with poor prognosis of cHCC-CC.

## Supplementary Material

Supplementary figures and tables.

## Figures and Tables

**Table 1:** Patients' baseline characteristics of cHCC-CC, HCC and ICC in the whole cohort prior to match

Variable	cHCC-CC (n=135)	HCC (n=698)	ICC (n=208)	*p*-value
**Sex, male, n (%)**				<0.001
Male	118 (87.4)	590 (84.5)	139 (66.8)	
Female	17 (12.6)	108 (15.5)	69 (33.2)	
**Age, year**				<0.001
≤60	105 (77.8)	547 (78.4)	124 (59.6)	
>60	30 (22.2)	151 (21.6)	84 (40.4)	
Hypertension, n (%)	13 (9.6)	65 (9.3)	35 (16.8)	0.008
Diabetes mellitus, n (%)	9 (6.7)	47 (6.7)	17 (8.2)	0.76
**Hepatitis B/C, n (%)**				<0.001
Present	68 (50.4)	516 (73.9)	90 (43.3)	
Absent	67 (49.6)	182 (26.1)	118 (56.7)	
Hypersplenia, n (%)	15 (11.1)	54 (7.7)	8 (3.8)	0.036
ALT, U/L, mean ± SD	53.6 ± 94.2	55.8 ± 65.5	41.6 ± 50.2	0.032
AST, U/L, mean ± SD	60.3 ± 129.4	62.2 ± 75.3	45.4 ± 48.5	0.032
ALB, g/L, mean ± SD	41.9 ± 4.7	40.3 ± 6.6	39.6 ± 9.4	0.011
TB, umol/L, mean ± SD	15.8 ± 9.8	16.1 ± 12.7	16.5 ± 18.3	0.90
PT, s, mean± SD	12.0 ± 1.9	12.5 ± 1.3	12.0 ± 1.4	<0.001
INR, mean± SD	1.0 ± 0.2	1.1 ± 0.1	1.0 ± 0.1	<0.001
AFP, ng/ml, mean ± SD	286.6 ± 476.0	449.5 ± 533.5	178.5 ± 407.5	<0.001
CA19-9, U/ml, mean ± SD	91.7 ± 223.1	32.6 ± 68.7	290.2 ± 391.9	<0.001
CA125,U/ml, mean ± SD	84.6 ± 523.3	35.3 ± 221.8	159.8 ± 578.9	0.011
CEA,ng/ml, mean ± SD	5.8 ± 28.1	3.1 ± 8.5	22.0 ± 97.9	<0.001
**Liver fibrosis, n (%)**				<0.001
No significant fibrosis	19 (14.1)	19 (3.1)	39 (18.8)	
Significant fibrosis	19 (14.1)	211 (33.9)	37 (17.8)	
Advanced fibrosis	42 (31.1)	75 (12.1)	97 (46.6)	
Liver cirrhosis	55 (40.7)	317 (51.0)	35 (16.8)	
**Tumor size, n (%)**				0.55
≤5 cm	44 (32.6)	195 (27.9)	57 (28.5)	
>5 cm	91 (67.4)	503 (72.1)	143 (71.5)	
**Tumor number, n (%)**				<0.001
Single	59 (43.7)	499 (71.5)	143 (68.8)	
Multiple	76 (56.3)	199 (28.5)	65 (31.2)	
Satellite lesions, n (%)	46 (34.1)	150 (21.5)	42 (20.2)	0.004
Tumor capsule, n (%)	23 (17.0)	116 (16.6)	82 (39.4)	<0.001
Tumor thrombus, n (%)	50 (37.0)	357 (51.1)	45 (21.6)	<0.001
Lymph node infiltration, n (%)	18 (13.3)	25 (3.6)	31 (14.9)	<0.001
**Differentiation, n (%)**				<0.001
Well	5 (3.7)	7 (1.1)	5 (2.4)	
Moderate	58 (43.0)	291 (47.4)	59 (28.4)	
Poor	61 (45.2)	313 (51.0)	135 (64.9)	
Undifferentiated	11 (8.1)	3 (0.5)	9 (4.3)	
**8^th^ AJCC stage, n (%)**				<0.001
I	11 (8.1)	275 (39.4)	76 (36.5)	
II	32 (23.7)	69 (9.9)	36 (17.3)	
III	74 (54.8)	338 (48.4)	65 (31.2)	
IV	18 (13.3)	16 (2.3)	31 (14.9)	
**T stage, n (%)**				<0.001
T1	15 (11.1)	279 (40.0)	85 (40.9)	
T2	35 (25.9)	69 (9.9)	39 (18.8)	
T3	52 (38.5)	64 (9.2)	45 (21.6)	
T4	33 (24.4)	286 (41.0)	39 (18.8)	
**N stage, n (%)**				<0.001
N0	117 (86.7)	673 (96.4)	177 (85.1)	
N1	18 (13.3)	25 (3.6)	31 (14.9)	
**M stage, n (%)**				NA
M0	135 (100.0)	698 (100.0)	208 (100.0)	
Transfusion				0.95
Yes	18 (13.3)	95 (14.0)	27 (13.2)	
No	117 (86.7)	586 (86.0)	178 (86.8)	
Blood loss, ≤400 ml, n (%)	80 (59.3)	469 (68.9)	148 (72.2)	0.036
**ASA, n (%)**	135	698	208	0.76
1	1 (2.1)	11 (3.5)	3 (2.6)	
2	43 (89.6)	242 (77.1)	88 (77.2)	
3	4 (8.3)	56 (17.8)	22 (19.3)	
4	0 (0.0)	4 (1.3)	1 (0.9)	
NA	87 (64.4)	385 (55.2)	95 (45.7)	
**Margin, n (%)**				<0.001
R0	112 (83.0)	603 (90.3)	139 (79.4)	
R1	23 (17.0)	65 (9.7)	36 (20.6)	
**Surgical method, n (%)**				<0.01
Major resection	61 (45.2)	339 (48.6)	137 (65.9)	
Minor resection	64 (47.4)	287 (41.1)	50 (24.0)	
Resection+ Ablation	7 (5.2)	60 (8.6)	18 (8.7)	
Liver transplantation	3 (2.2)	12 (1.7)	3 (1.4)	
Anatomy resection, n (%)	57 (43.8)	271 (40.9)	139 (71.3)	<0.001
**Postoperative TACE, n (%)**				<0.001
Yes	40 (29.6)	326 (46.7)	80 (38.5)	
No	95 (70.4)	372 (53.3)	128 (61.5)	
Disease-free survival, m, mean± SD	28.9± 28.2	21.8±17.1	12.1±16.0	<0.001
Overall survival, m, mean± SD	32.3±27.4	28.2±18.5	15.7±16.0	<0.001

Abbreviation: cHCC-CC: Combined hepatocellular carcinoma and cholangiocarcinoma; HCC: hepatocellular carcinoma; ICC: intrahepatic cholangiocarcinoma; ALT: alanine aminotransferase; SD: standard deviation; AST: aspartate aminotransferase; ALB: albumin; TB: total bilirubin; PT: prothrombin time; INR: International Normalized Ratio; AFP: alpha fetoprotein; CEA: carcinoembryonic antigen; AJCC: American Joint Committee on Cancer; ASA: American Society of Anesthesiology; NA: not applicable; TACE: transhepatic arterial chemotherapy and embolization; ref: reference.

**Table 2:** Univariate and multivariate analysis of overall survival prior to match

Variable	Univariate		Multivariate	
	HR (95% CI)	*p*-value	HR (95% CI)	*p*-value
Sex, male	1.1 (0.9, 1.3)	0.410		
ALB, g/L	1.0 (1.0, 1.0)	0.005	1.0 (1.0, 1.0)	0.013
**Liver fibrosis**				
No significant fibrosis	Ref		Ref	
Significant fibrosis	1.4 (1.0, 2.0)	0.064	1.7 (1.2, 2.6)	0.008
Advanced fibrosis	1.7 (1.2, 2.4)	0.002	2.1 (1.4, 3.1)	<0.001
Liver cirrhosis	1.5 (1.0, 2.2)	0.036	1.4 (0.9, 2.2)	0.124
Tumor size, >5 cm	1.7 (1.4, 2.0)	<0.001	1.3 (1.0, 1.6)	0.034
Tumor number, ≥2	1.2 (1.0, 1.4)	0.019	1.0 (0.7, 1.3)	0.825
Satellite lesions, absent	0.7 (0.6, 0.8)	<0.001	0.8 (0.6, 1.1)	0.203
Tumor thrombus, absent	0.7 (0.6, 0.8)	<0.001	0.8 (0.6, 1.0)	0.080
**Lymph node infiltration**				
Present	Ref		Ref	
Absent	2.0 (1.67, 2.5)	<0.001	2.5 (1.1, 5.0)	0.021
**Differentiation**				
Well	Ref		Ref	
Moderate	2.9 (1.1, 7.7)	0.037	3.4 (1.1, 10.7)	0.039
Poor	5.6 (2.1, 14.9)	<0.001	5.8 (1.8, 18.3)	0.003
Undifferentiated	19.7 (6.7, 58.0)	<0.001	22.8 (6.4, 81.6)	<0.001
**8^th^ AJCC stage**				
I	Ref		Ref	
II	0.8 (0.6, 1.1)	0.172	0.6 (0.4, 0.9)	0.022
III	1.8 (1.5, 2.1)	<0.001	1.4 (1.0, 2.1)	0.044
IV	2.8 (2.1, 3.8)	<0.001	2.6 (1.9, 3.7)	<0.001
Transfusion, yes	0.6 (0.5, 0.8)	<0.001	0.9 (0.7, 1.2)	0.343
Blood loss, >400 ml	1.4 (1.2, 1.6)	<0.001	1.2 (1.0, 1.5)	0.059
Margin, R1	1.7 (1.4, 2.2)	<0.001	1.7 (1.3, 2.1)	<0.001
Anatomy resection	0.8 (0.7, 0.9)	0.002	0.9 (0.7, 1.1)	0.277
Postoperative TACE	1.8 (1.6, 2.2)	<0.001	1.8 (1.5, 2.2)	<0.001
**Tumor type**				
cHCC-CC	Ref		Ref	
HCC	1.0 (0.8, 1.2)	0.799	0.9 (0.6, 1.2)	0.397
ICC	2.3 (1.8, 3.1)	<0.001	3.5 (2.5, 4.9)	<0.001

Abbreviation: cHCC-CC: Combined hepatocellular carcinoma and cholangiocarcinoma; HCC: hepatocellular carcinoma; ICC: intrahepatic cholangiocarcinoma; HR: hazard ratio; CI: confidence interval; Ref: reference; ALB: albumin; AJCC: American Joint Committee on Cancer; TACE: transhepatic arterial chemotherapy and embolization; HR: hazard ratio; CI: confidence interval; ref: reference.

**Table 3:** Comparison among Patients with cHCC-CC, HCC and ICC when matched on ALB, tumor size, lymph node infiltration, AJCC stage and margin

Variable	1:1 match			1:1 match		
	cHCC-CC (n=131)	HCC (n=131)	*p*-value	cHCC-CC (n=109)	ICC (n=109)	*p*-value
Sex, male, n (%)	114 (87)	111 (84.7)	0.72	95 (87.2)	69 (63.3)	<0.01
Age, year, ≤60	102 (77.9)	110 (84)	0.27	83 (76.1)	71 (65.1)	0.10
Hypertension, n (%)	13 (9.9)	12 (9.2)	1.00	10 (9.2)	16 (14.7)	0.30
Diabetes mellitus, n (%)	9 (6.9)	9 (6.9)	1.00	7 (6.4)	4 (3.7)	0.54
Hepatitis, n (%)	66 (50.4)	95 (72.5)	0.0004	55 (50.5)	49 (45)	0.50
Hypersplenia, n (%)	14 (10.7)	14 (10.7)	1.00	12 (11)	7 (6.4)	0.34
ALT, U/L, mean ± SD	54.37 ± 95.48	58.22 ± 60.64	0.70	47.25 ± 34.64	47.57 ± 63.21	0.96
AST, U/L, mean ± SD	61.13 ± 131.24	62.10 ± 58.04	0.94	49.97 ± 38.71	50.13 ± 60.81	0.98
ALB, g/L, mean ± SD	41.9 ± 4.6	41.9 ± 4.2	0.98	41.7 ± 4.4	39.8 ± 8.4	0.93
TB, umol/L, mean ± SD	15.86 ± 9.84	15.49 ± 10.07	0.77	15.97 ± 9.71	18.54 ± 22.93	0.28
PT, s, mean± SD	12.01 ± 1.91	12.34 ± 1.40	0.18	11.99 ± 1.92	12.07 ± 1.62	0.79
INR, mean± SD	1.04 ± 0.16	1.07 ± 0.12	0.15	1.04 ± 0.16	1.04 ± 0.14	0.82
AFP, ng/ml, mean ± SD	231.6 ± 406.0	440.5 ± 433.5	0.002	106.08 ± 245.92	311.55 ± 406.11	<0.0001
Ca19-9, U/ml, mean ± SD	92.81 ± 226.09	23.99 ± 26.21	0.0021	100.11 ± 579.37	94.91 ± 252.25	0.95
Ca125,U/ml, mean ± SD	87.10 ± 531.45	74.81 ± 465.68	0.87	6.53 ± 31.29	24.78 ± 114.66	0.12
CEA, ng/ml, mean ± SD	5.92 ± 28.51	4.21 ± 17.38	0.59	47.25 ± 34.64	47.57 ± 63.21	0.9
**Liver fibrosis, n (%)**			<0.0001			0.0045
No significant fibrosis	19 (14.5)	3 (2.5)		16 (14.7)	18 (16.5)	
Significant fibrosis	19 (14.5)	42 (35.3)		15 (13.8)	20 (18.3)	
Advanced fibrosis	41 (31.3)	64 (53.8)		33 (30.3)	50 (45.9)	
liver cirrhosis	52 (39.7)	10 (8.4)		45 (41.3)	21 (19.3)	
Tumor size, >5cm, n (%)	87 (66.4)	101 (77.1)	0.074	69 (63.3)	74 (71.2)	0.28
Tumor number, single, n (%)	59 (45)	68 (51.9)	0.32	52 (47.7)	65 (59.6)	0.10
Satellite lesions, n (%)	44 (33.6)	44 (33.6)	1.00	34 (31.2)	27 (24.8)	0.37
Tumor capsule, n (%)	23 (17.6)	29 (22.1)	0.37	18 (16.5)	35 (32.1)	<0.0001
Tumor thrombus, n (%)	47 (35.9)	92 (70.2)	<0.0001	34 (31.2)	30 (27.5)	0.66
Lymph node infiltration, n (%)	14 (10.7)	14 (10.7)	1.00	18 (16.5)	18 (16.5)	1.00
**Differentiation, n (%)**			0.0028			0.016
Well	5 (3.8)	1 (0.8)		4 (3.7)	4 (3.7)	
Moderate	55 (42)	57 (45.2)		48 (44)	26 (23.9)	
Poor	60 (45.8)	68 (54)		54 (49.5)	73 (67)	
Undifferentiated	11 (8.4)	0 (0)		3 (2.8)	6 (5.5)	
**8^th^ AJCC stage, n (%)**			1.00			1.00
I	11 (8.4)	11 (8.4)		11 (10.1)	11 (10.1)	
II	32 (24.4)	32 (24.4)		32 (29.4)	32 (29.4)	
III	74 (56.5)	74 (56.5)		48 (44)	48 (44)	
IV	14 (10.7)	14 (10.7)		18 (16.5)	18 (16.5)	
**T stage, n (%)**			0.013			0.76
T1	15 (11.5)	14 (10.7)		15 (13.8)	16 (14.7)	
T2	35 (26.7)	32 (24.4)		35 (32.1)	32 (29.4)	
T3	48 (36.6)	29 (22.1)		39 (35.8)	35 (32.1)	
T4	33 (25.2)	56 (42.7)		20 (18.3)	26 (23.9)	
**N stage, n (%)**			1.00			1.00
N0	117 (89.3)	117 (89.3)		91 (83.5)	91 (83.5)	
N1	14 (10.7)	14 (10.7)		18 (16.5)	18 (16.5)	
**Transfusion, n (%)**	18 (13.7)	11 (8.7)	0.27	16 (14.7)	9 (8.5)	0.23
Blood loss, ≤400ml, n (%)	77 (58.8)	90 (70.9)	0.057	66 (60.6)	84 (79.2)	0.0046
Margin, R0, n (%)	108 (82.4)	119 (91.5)	0.046	87 (79.8)	62 (70.5)	0.18
Surgical method, n (%)			0.0772			0.0003
Major resection	59 (45)	73 (55.7)		53 (48.6)	68 (62.4)	
Minor resection	63 (48.1)	44 (33.6)		50 (45.9)	25 (22.9)	
Resection+ Ablation	6 (4.6)	11 (8.4)		4 (3.7)	15 (13.8)	
Liver transplantation	3 (2.3)	3 (2.3)		2 (1.8)	1 (0.9)	
Anatomy resection, n (%)	54 (42.9)	66 (52.4)	0.17	49 (46.7)	72 (72.7)	0.0003
Postoperative TACE, n (%)	39 (29.8)	83 (63.4)	<0.0001	31 (28.4)	39 (35.8)	0.31
Disease-free survival, m, mean± SD	28.5± 28.1	22.4± 13.1	0.025	25.7 ± 25.8	9.3 ± 12.6	<0.0001
Overall survival, m, mean± SD	31.9± 27.2	33.3± 18.1	0.62	29.4 ± 25.3	13.7 ± 12.9	<0.0001

Abbreviation: cHCC-CC: Combined hepatocellular carcinoma and cholangiocarcinoma; HCC: hepatocellular carcinoma; ICC: intrahepatic cholangiocarcinoma; ALT: alanine aminotransferase; SD: standard deviation; AST: aspartate aminotransferase; ALB: albumin; TB: total bilirubin; PT: prothrombin time; INR: International Normalized Ratio; AFP: alpha fetoprotein; CEA: carcinoembryonic antigen; AJCC: American Joint Committee on Cancer; ASA: American Society of Anesthesiology; NA: not applicable; TACE: transhepatic arterial chemotherapy and embolization.

**Table 4:** Univariate and multivariate analysis of overall survival of two matched cohorts

Variable	1:1 match of cHCC-CC (n=131) and HCC (n=131)			1:1 match of cHCC-CC (n=109) and ICC (n=109)		
	Univariate: *p* value	Multivariate: HR (95% CI)	*p*-value	Univariate: *p* value	Multivariate: HR (95% CI)	*p*-value
Sex, male	0.197			0.994		
ALB, g/L	0.991			0.554		
**Tumor size**						
≤5 cm	Ref			Ref	Ref	Ref
>5 cm	0.949			<0.001	1.3 (0.8, 2.3)	0.288
Satellite lesions	0.216			<0.001	0.8 (0.4, 1.4)	0.405
Tumor thrombus	0.816			<0.001	0.7 (0.4, 1.2)	0.161
**Lymph node infiltration**						
Negative	Ref	Ref	Ref	Ref	Ref	Ref
Positive	<0.001	2.5 (1.1, 5.0)	0.025	0.029	1.7 (1.1, 2.5)	0.028
**Differentiation**						
Well	Ref	Ref	Ref	Ref	Ref	Ref
Moderate	0.594	2.5 (0.3, 18.8)	0.378	0.305	2.2 (0.3, 16.9)	0.459
Poor	0.090	8.2 (1.1, 61.3)	0.040	0.012	3.8 (0.5, 29.8)	0.203
Undifferentiated	0.002	35.0 (4.3, 282.0)	<0.001	0.013	3.4 (0.1, 78.2)	0.441
**8^th^ AJCC stage**						
I	Ref	Ref	Ref	Ref	Ref	Ref
II	0.097	0.5 (0.2, 1.1)	0.084	0.933	1.7 (0.5, 5.7)	0.367
III	0.801	1.3 (0.6, 2.8)	0.480	0.015	2.3 (0.7, 7.3)	0.164
IV	0.018	2.6 (1.1, 6.0)	0.025	0.014	2.9 (0.9, 9.7)	0.082
Margin, R1	0.020	2.3 (1.3, 4.1)	0.005	0.755	0.8 (0.4, 1.5)	0.466
**Surgical method**						
Major resection	Ref	Ref	Ref	Ref	Ref	Ref
Minor resection	0.061	0.6 (0.4, 0.9)	0.015	0.002	0.9 (0.5, 1.8)	0.829
Resection+ Ablation	0.033	2.2 (0.6, 7.7)	0.237	0.109	0.8 (0.2, 3.5)	0.720
Liver transplantation	0.532	0.3 (0.1, 1.4)	0.127	0.322	0.4 (0.1, 2.7)	0.997
**Postoperative TACE**						
Yes	Ref	Ref	Ref	Ref	Ref	Ref
No	<0.001	2.8 (1.8, 4.3)	<0.001	<0.001	2.5 (1.4, 4.5)	0.003
**Tumor type**						
cHCC-CC	Ref	Ref	Ref	Ref	Ref	Ref
HCC	<0.001	0.3 (0.2, 0.6)	<0.001	-	-	-
ICC	-	-	-	0.061	1.3 (0.9, 2.0)	0.129

Abbreviation: cHCC-CC: Combined hepatocellular carcinoma and cholangiocarcinoma; HCC: hepatocellular carcinoma; ICC: intrahepatic cholangiocarcinoma; HR: hazard ratio; CI: confidence interval; Ref: reference; ALB: albumin; AJCC: American Joint Committee on Cancer; TACE: transhepatic arterial chemotherapy and embolization; HR: hazard ratio; CI: confidence interval; ref: reference.

## Abbreviations

cHCC-CC: Combined hepatocellular carcinoma and cholangiocarcinoma
HCC: hepatocellular carcinoma
ICC: intrahepatic cholangiocarcinoma
WHO: World Health Organization
CLC: cholangiolocellular carcinoma
OS: overall survival
DFS: disease-free survival
HR: hazard ratio
CI: confidence interval
Ref: reference
ALT: alanine aminotransferase
SD: standard deviation
AST: aspartate aminotransferase
ALB: albumin
TB: total bilirubin
PT: prothrombin time
INR: International Normalized Ratio
AFP: alpha fetoprotein
CEA: carcinoembryonic antigen
AJCC: American Joint Committee on Cancer
LN: lymph node
TACE: transhepatic arterial chemotherapy and embolization
