# Supplementary material for: The clinical characteristics and prognostic factors of combined Hepatocellular Carcinoma and Cholangiocarcinoma, Hepatocellular Carcinoma and Intrahepatic Cholangiocarcinoma after Surgical Resection: A propensity score matching analysis
Source: Int J Med Sci. 2021 Jan 1;18(1):187–98. doi: 10.7150/ijms.50883 (PMC7738961; doi:10.7150/ijms.50883)
Supplement: Supplementary file 1 — Supplementary figures and tables. [file ijmsv18p0187s1.pdf]

Supplement Figure 1 Survival curves of cHCC-CC in unmatched cohorts stratified by LN infiltration and postoperative TACE. A and B: The OS and DFS in cHCC-CC stratified by LN infiltration. C and D: The OS and DFS in cHCC-CC stratified by postoperative TACE. Patient with negative LN infiltration or receiving postoperative TACE obtain favorable OS and DFS than those who have positive LN or didn't receive postoperative TACE (p<0.05). LN: lymph node; TACE: transhepatic arterial chemotherapy and embolization; OS: overall survival; DFS: disease-free survival; cHCC-CC: Combined hepatocellular carcinoma and cholangiocarcinoma; HCC: hepatocellular carcinoma; ICC: intrahepatic cholangiocarcinoma;

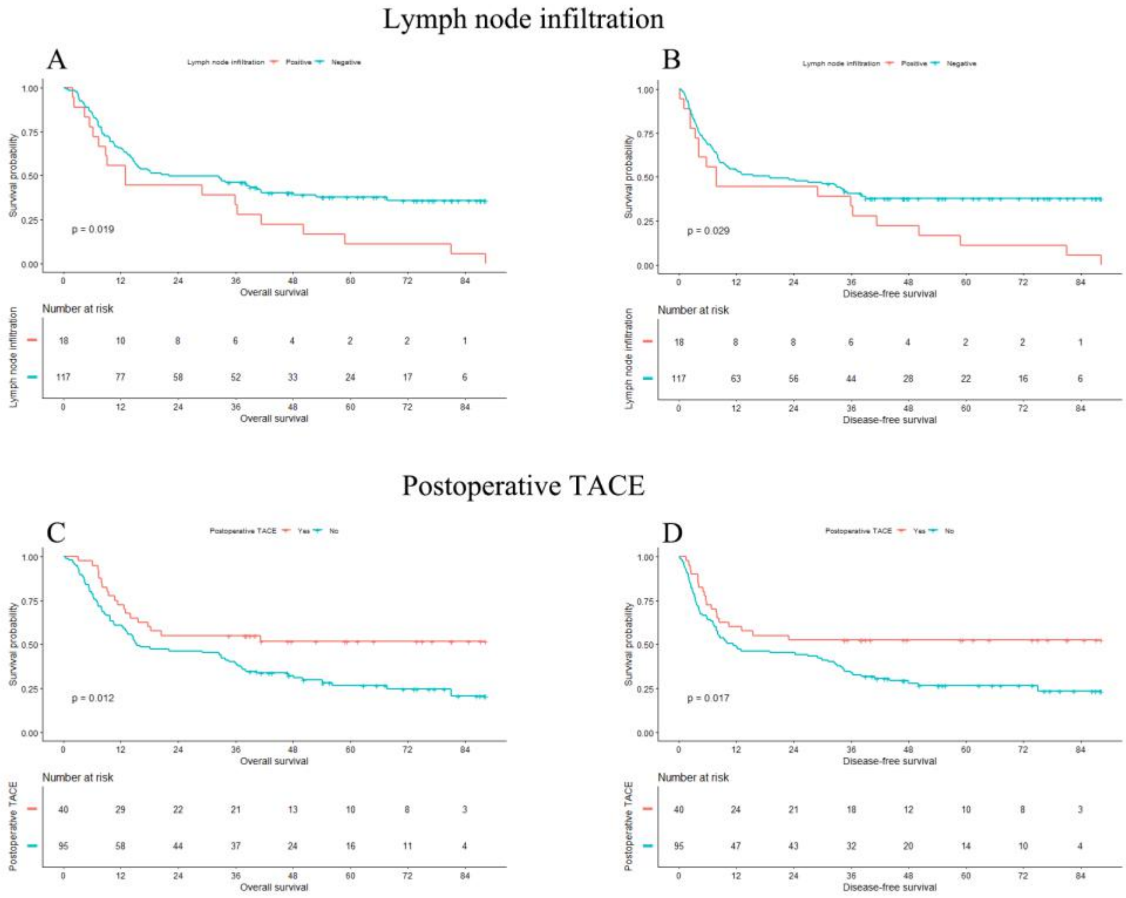

Supplement Table 1 Details of Univariate and multivariate analysis of overall survival prior to match. (including tumor size and T stage, lymph node infiltration and N stage)

Variable	Univariate		Multivariate	
	HR (95% CI)	p-value	HR (95% CI)	p-value
Sex, male	1.1 (0.9, 1.3)	0.410		
Age, >60 year	1.0 (0.9, 1.2)	0.711		
Hypertension	0.9 (0.7, 1.1)	0.208		
Diabetes mellitus,	0.9 (0.7, 1.2)	0.451		
Hepatitis	1.1 (0.9, 1.3)	0.278		
Hypersplenia, n (%)	1.0 (0.7, 1.3)	0.942		
ALT, U/L	1.0 (1.0, 1.0)	0.160		
AST, U/L	1.0 (1.0, 1.0)	0.006	1.0 (1.0, 1.0)	0.077

ALB, g/L	1.0 (1.0, 1.0)	0.005	1.0 (0.9, 1.0)	0.046
TB, umol/L	1.0 (1.0, 1.0)	0.021	1.0 (1.0, 1.0)	0.461
PT, s	1.1 (1.0, 1.1)	0.042	1.2 (0.9, 1.7)	0.243
INR	2.0 (1.0, 4.0)	0.050	0.3 (0.0, 17.4)	0.588
AFP, >=400ng/ml	1.1 (0.9, 1.3)	0.309		
CA 19-9, U/ml	1.0 (1.0, 1.0)	<0.001	1.0 (1.0, 1.0)	0.990
CA 125,U/ml	1.0 (1.0, 1.0)	<0.001	1.0 (1.0, 1.0)	0.937
CEA, ng/ml	1.0 (1.0, 1.0)	<0.001	1.0 (1.0, 1.0)	0.793
Liver fibrosis				
No significant fibrosis	Ref		Ref	
Significant fibrosis	1.4 (1.0, 2.0)	0.064	1.3 (0.9, 1.8)	0.206
Advanced fibrosis	1.7 (1.2, 2.4)	0.002	1.6 (1.1, 2.3)	0.010
liver cirrhosis	1.5 (1.0, 2.2)	0.036	1.2 (0.8, 1.8)	0.345
Tumor size				
≤5cm	Ref		Ref	
＞5cm	1.7 (1.4, 2.0)	<0.001	1.5 (1.2, 1.8)	<0.001
Tumor number, ≥2	1.2 (1.0, 1.4)	0.019	1.0 (0.8, 1.3)	0.793
Satellite lesions				
Present	Ref		Ref	
Absent	0.7 (0.6, 0.8)	<0.001	0.8 (0.6, 1.0)	0.090
Tumor capsule	1.2 (0.9, 1.4)	0.209		
Tumor thrombus				
Present	Ref		Ref	
Absent	0.7 (0.6, 0.8)	<0.001	0.9 (0.7, 1.0)	0.107
Lymph node infiltration				
Present	Ref		Ref	
Absent	2.0 (1.7, 2.5)	<0.001	1.7(1.4, 2.5)	<0.001
Differentiation				
Well	Ref		Ref	
Moderate	2.9 (1.1, 7.7)	0.037	2.3 (0.8, 6.2)	0.103
Poor	5.6 (2.1, 14.9)	<0.001	4.2 (1.5, 11.4)	0.005
Undifferentiated	19.7 (6.7, 58.0)	<0.001	20.9 (7.0, 62.2)	<0.001
8th AJCC stage				
I	Ref		Ref	
II	0.8 (0.6, 1.1)	0.172	0.7 (0.5, 1.0)	0.070
III	1.8 (1.5, 2.1)	<0.001	1.8 (1.5, 2.2)	<0.001
IV	2.8 (2.1, 3.8)	<0.001	2.6 (1.9, 3.7)	<0.001
T stage				
T1	Ref		Ref	
T2	0.8 (0.6, 1.1)	0.246	3.1 (1.0, 9.2)	0.042

T3	1.6 (1.3, 2.1)	<0.001	1.6 (0.8, 3.2)	0.200
T4	1.9 (1.5, 2.2)	<0.001	1.8 (0.9, 3.6)	0.096
N stage				
N0	Ref		Ref	
N1	2.2 (1.7, 2.8)	<0.001	0.9 (0.7, 1.2)	0.426
Transfusion, no	0.6 (0.5, 0.8)	<0.001	0.8 (0.6, 1.0)	0.094
Blood loss, >400ml	1.4 (1.2, 1.6)	<0.001	1.2 (1.0, 1.4)	0.094
Margin, R1	1.7 (1.4, 2.2)	<0.001	1.7 (1.3, 2.1)	<0.001
Surgical method				
Major resection	Ref		Ref	
Minor resection	0.7 (0.6, 0.8)	<0.001	0.8 (0.6, 0.9)	0.005
Resection+ Ablation	0.8 (0.5, 1.3)	0.432	0.8 (0.5, 1.5)	0.556
Liver transplantation	0.8 (0.4, 1.4)	0.383	0.5 (0.2, 1.0)	0.054
Anatomy resection	0.8 (0.7, 0.9)	0.002	0.9 (0.7, 1.1)	0.277
Postoperative TACE	1.8 (1.6, 2.2)	<0.001	2.3 (1.6, 3.4)	<0.001
Tumor type				
cHCC-CC	Ref		Ref	
HCC	1.0 (0.8, 1.2)	0.799	1.1 (0.8, 1.4)	0.720
ICC	2.3 (1.8, 3.1)	<0.001	3.5 (2.6, 4.8)	<0.001

Abbreviation: HR: hazard ratio; CI: confidence interval; ref: reference; cHCC-CC: Combined hepatocellular carcinoma and cholangiocarcinoma; HCC: hepatocellular carcinoma; ICC: intrahepatic cholangiocarcinoma; ALT: alanine aminotransferase; AST: aspartate aminotransferase; ALB: albumin; TB: total bilirubin; PT: prothrombin time; INR: International Normalized Ratio; AFP: alpha fetoprotein; CEA: carcinoembryonic antigen; AJCC: American Joint Committee on Cancer; TACE: transhepatic arterial chemotherapy and embolization.

Supplement Table 2. Univariate and multivariate analysis of overall survival prior to match. (only including tumor size and N stage)

Variable	Univariate		Multivariate	
	HR (95% CI)	p-value	HR (95% CI)	p-value
N stage				
N0	Ref		Ref	
N1	2.2 (1.7, 2.8)	<0.001	1.8 (1.4, 2.4)	<0.001
Sex, male	1.1 (0.9, 1.3)	0.410	0.9 (0.7, 1.1)	0.261
ALT, U/L	1.0 (1.0, 1.0)	0.160		
AST, U/L	1.0 (1.0, 1.0)	0.006	1.0 (1.0, 1.0)	0.077
ALB, g/L	1.0 (1.0, 1.0)	0.005	1.0 (1.0, 1.0)	0.013
TB, umol/L	1.0 (1.0, 1.0)	0.021	1.0 (1.0, 1.0)	0.461
PT, s	1.1 (1.0, 1.1)	0.042	1.2 (0.9, 1.7)	0.243
INR	2.0 (1.0, 4.0)	0.050	0.3 (0.0, 17.4)	0.588
AFP				

＜400 ng/ml	Ref			
≥400 ng/ml	1.1 (0.9, 1.3)	0.309		
CA 19-9, U/ml	1.0 (1.0, 1.0)	<0.001	1.0 (1.0, 1.0)	0.990
CA 125,U/ml	1.0 (1.0, 1.0)	<0.001	1.0 (1.0, 1.0)	0.937
CEA, ng/ml	1.0 (1.0, 1.0)	<0.001	1.0 (1.0, 1.0)	0.793
Liver fibrosis				
No significant fibrosis	Ref		Ref	
Significant fibrosis	1.4 (1.0, 2.0)	0.064	1.7 (1.2, 2.6)	0.008
Advanced fibrosis	1.7 (1.2, 2.4)	0.002	2.1 (1.4, 3.1)	<0.001
liver cirrhosis	1.5 (1.0, 2.2)	0.036	1.4 (0.9, 2.2)	0.124
Tumor size, >5cm	1.7 (1.4, 2.0)	<0.001	1.3 (1.0, 1.6)	<0.001
Tumor number, ≥2	1.2 (1.0, 1.4)	0.019	1.0 (0.7, 1.3)	0.825
Satellite lesions, absent	0.7 (0.6, 0.8)	<0.001	0.8 (0.6, 1.1)	0.203
Tumor thrombus, absent	0.7 (0.6, 0.8)	<0.001	0.8 (0.6, 1.0)	0.080
Differentiation				
Well	Ref		Ref	
Moderate	2.9 (1.1, 7.7)	0.037	3.4 (1.1, 10.7)	0.039
Poor	5.6 (2.1, 14.9)	<0.001	5.8 (1.8, 18.3)	0.003
Undifferentiated	19.7 (6.7, 58.0)	<0.001	22.8 (6.4, 81.6)	<0.001
8th AJCC stage				
I	Ref		Ref	
II	0.8 (0.6, 1.1)	0.172	0.6 (0.4, 0.9)	0.022
III	1.8 (1.5, 2.1)	<0.001	1.4 (1.0, 2.1)	0.044
IV	2.8 (2.1, 3.8)	<0.001	0.7 (0.3, 1.8)	0.426
Transfusion				
Yes	Ref		Ref	
No	0.6 (0.5, 0.8)	<0.001	0.9 (0.7, 1.2)	0.343
Blood loss, >400ml	1.4 (1.2, 1.6)	<0.001	1.2 (1.0, 1.5)	0.059
Margin, R1	1.7 (1.4, 2.2)	<0.001	1.4 (1.1, 1.9)	0.005
Anatomy resection	0.8 (0.7, 0.9)	0.002	0.9 (0.7, 1.1)	0.277
Postoperative TACE	1.8 (1.6, 2.2)	<0.001	1.8 (1.5, 2.2)	<0.001
Tumor type				
cHCC-CC	Ref		Ref	
HCC	1.0 (0.8, 1.2)	0.799	0.9 (0.6, 1.2)	0.397
ICC	2.3 (1.8, 3.1)	<0.001	3.5 (2.5, 4.9)	<0.001

Abbreviation: HR: hazard ratio; CI: confidence interval; ref: reference; cHCC-CC: Combined hepatocellular carcinoma and cholangiocarcinoma; HCC: hepatocellular carcinoma; ICC: intrahepatic cholangiocarcinoma; ALT: alanine aminotransferase; AST: aspartate aminotransferase; ALB: albumin; TB: total bilirubin; PT: prothrombin time; INR: International Normalized Ratio; AFP: alpha fetoprotein; CEA: carcinoembryonic antigen; AJCC: American Joint Committee on Cancer; TACE: transhepatic arterial chemotherapy and embolization.

Supplement Table 3. Power analysis of the study.

**cHCC/ ICC**	value	**cHCC/ HCC**	Value
Two-sided α	0.05	Two-sided α	0.05
5-year overall survival rate (cHCC-CC)	0.395	5-year overall survival rate (cHCC-CC)	0.395
5-year overall survival rate (ICC)	0.179	5-year overall survival rate (HCC)	0.179
Assuming hazard ratio	0.737	Assuming hazard ratio	0.838
Power	90%	Power	90%
Estimated events (cHCC-CC)	56	Estimated events (cHCC-CC)	128
Estimated events (ICC)	56	Estimated events (ICC)	511

Abbreviation: cHCC-CC: Combined hepatocellular carcinoma and cholangiocarcinoma; HCC: hepatocellular carcinoma; ICC: intrahepatic cholangiocarcinoma;
